# Effects of Facial Symmetry and Gaze Direction on Perception of Social Attributes: A Study in Experimental Art History

**DOI:** 10.3389/fnhum.2016.00452

**Published:** 2016-09-13

**Authors:** Per O. Folgerø, Lasse Hodne, Christer Johansson, Alf E. Andresen, Lill C. Sætren, Karsten Specht, Øystein O. Skaar, Rolf Reber

**Affiliations:** ^1^Department of Linguistic, Literary and Aesthetic Studies, University of BergenBergen, Norway; ^2^Department of Art and Media Studies, Norges Teknisk-Naturvitenskapelige Universitet (NTNU)Trondheim, Norway; ^3^Department of Biological and Medical Psychology, University of BergenBergen, Norway; ^4^Department of Psychology, University of OsloOslo, Norway

**Keywords:** experimental aesthetics, emotion, face perception, gaze perception, holy face

## Abstract

This article explores the possibility of testing hypotheses about art production in the past by collecting data in the present. We call this enterprise “experimental art history”. Why did medieval artists prefer to paint Christ with his face directed towards the beholder, while profane faces were noticeably more often painted in different degrees of profile? Is a preference for frontal faces motivated by deeper evolutionary and biological considerations? Head and gaze direction is a significant factor for detecting the intentions of others, and accurate detection of gaze direction depends on strong contrast between a dark iris and a bright sclera, a combination that is only found in humans among the primates. One uniquely human capacity is language acquisition, where the detection of shared or joint attention, for example through detection of gaze direction, contributes significantly to the ease of acquisition. The perceived face and gaze direction is also related to fundamental emotional reactions such as fear, aggression, empathy and sympathy. The fast-track modulator model presents a related fast and unconscious subcortical route that involves many central brain areas. Activity in this pathway mediates the affective valence of the stimulus. In particular, different sub-regions of the amygdala show specific activation as response to gaze direction, head orientation and the valence of facial expression. We present three experiments on the effects of face orientation and gaze direction on the judgments of social attributes. We observed that frontal faces with direct gaze were more highly associated with positive adjectives. Does this help to associate positive values to the Holy Face in a Western context? The formal result indicates that the Holy Face is perceived more positively than profiles with both direct and averted gaze. Two control studies, using a Brazilian and a Dutch database of photographs, showed a similar but weaker effect with a larger contrast between the gaze directions for profiles. Our findings indicate that many factors affect the impression of a face, and that eye contact in combination with face direction reinforce the general impression of portraits, rather than determine it.

## Introduction

In this article, we examine the possibility to test hypotheses about art production in the past by collecting data in the present. Similar work has gained recent attention in experimental archeology (e.g., Coles, 2010; Ferguson, 2010), where hypotheses about the past are investigated through the production and use of tools and objects in the present. We are not as concerned with the technical aspects of art production *per se*, but the plausible detection of artistic intentions and reception of artworks in the past. Specifically, we examine basic perceptual processes that presumably have not changed significantly over the centuries. Artistic norms might counteract these basic perceptual processes, but it is more likely that they are in line with basic perceptual and emotional processes and biases. Our contribution provides an example of how interdisciplinary research that includes art historians and psychologists might address a question from Western Medieval and Renaissance art by means of psychological experiments. This endeavor is called “experimental art history”.

We start with the observation that in this period the majority of portraits of Christ were frontal with a gaze directed toward the beholder. In this context, we define portrait as an image of one person alone showing only his/her face or the upper part of the body, painted on canvas or wooden support. The representations of identifiable persons in larger compositions, group portraits or narrative settings are not considered in our study. The frontal portraits (with very few exceptions) represent Christ as God, i.e., what is labeled the Holy Face (as opposed to the profiles, where He is the sufferer, the Man of Sorrows). This study looks at Christ as God and will exclude the Man of Sorrows. Previous studies have demonstrated that in the 15th and 16th centuries, almost all profane portraits (in contrast to the depictions of Christ) were depicted in different degrees of profile, very seldom in frontal view (Hodne, 2013). Famous exceptions were Albrecht Dürer and Hans Holbein the Younger. We notice the same tendency in later periods as well. Why did these artists prefer to paint Christ with his face directed towards the beholder, while profane faces were noticeably more often painted in different degrees of profile? Art historians usually take recourse to historical sources in order to answer such questions. There is a strong tradition in the West of copying the veil of Veronica as a template for the face of Christ. The blood and sweat on the relic was thought to be imprinted on the veil directly from the face of Christ by blood wiped from his face during his way to Golgotha. According to tradition, the intensity of Christ’s gaze in the veil made it necessary to cover the relic with a piece of cloth. The highly venerated relic was kept in the Old St Peter’s basilica (Rome). This symmetrical face with the strong direct gaze became the standard way to represent the Holy Face in Western art of the Renaissance both north and south of the Alps (Morgan, 2012: p. 55–62). But could there not be other reasons for the strong preference of full frontal portraits with a directed gaze? Such reasons could point to factors deep in human emotional responses to face perception.

We wanted to find out whether convention is the only answer to the Renaissance preference for representing faces of the holy in frontal view. Can we exclude that preference for frontal faces in the depiction of deity may have deeper *evolutionary* and* biological* reasons? Can there be other and *biologically* driven mechanisms leading to a preference for the strongly symmetrical face (i.e., the portrait in frontal view, as opposed to half-profile) for holy persons, and can this answer the question why Christ is depicted in frontal view and profane faces in half-profile?

We develop the idea that experimental art history can examine the plausibility of different hypotheses by assessing the effect of different face and gaze directions on modern viewers of portraits. Hence, are there deeper reasons for painting Christ in frontal view and profane faces in half-profile? Surely these masters were able to paint a face in whatever orientation they liked, so the possibility that the painter’s competence accounts for the result is not a valid explanation. We instead assume that the difference stems from the differential effects artists wanted to achieve through using our perceptual and emotional biases in face perception. However, as the short review of the reception and meaning of the veil of Veronica shows, the prototype became the paradigm about how the Holy Face should be represented. But could it have stayed the prototype for so long, if it had no other support?

Moving now to modern psychology, both the literature about gaze and the knowledge about the activation in the brain, in response to both face direction and gaze direction, has expanded tremendously during the last few years. A fast capture of head and gaze direction is a significant factor for detecting the other’s intention (Emery, 2000), and it must have represented a strong selective pressure during evolution. The accurate detection of gaze direction depends on the great contrast between the dark iris and the bright sclera. Kobayashi and Koshima (1997) found that among 88 primate species, only humans had eyes with a white sclera and a dark iris. In language acquisition, the detection of shared or joint attention, at the lowest level through detection of gaze direction, “may be important for language learning in human infants” (Emery, 2000, pp. 588; see also Tomasello and Farrar, 1986; Dunham et al., 1993; Mundi and Gomes, 1998). This illustrates the importance of gaze direction in human evolution, with precursors in other primates (see Emery, 2000, particularly pp. 584–587).

Researchers have focused on the perception of gaze direction and facial expression under different head orientations, (e.g., Langton, 2000; Todorović, 2006; Todorović, 2009; Ewbank et al., 2009; see also Wollaston, 1824). Advanced single cell recordings from the temporal cortex of macaque monkeys yield important data on the neuronal correlates to different directions of eye gaze, and head orientation, laterally as well as vertically (Perrett et al., 1985, 1992).

People who look directly at their counterpart could signal aggression or superiority, they could want to monitor the other’s actions, but they could also express the wish to communicate with or to care for their counterpart. This activates the brain’s so called Theory of Mind (ToM)-network, which is the social network through which individuals analyze another person’s intentions (e.g., Perrett and Emery, 1994; Baron-Cohen, 1997; Conty et al., 2007; Yang et al., 2015). The five main anatomical hotspots in the ToM network are *the superior temporal sulcus* (Perrett et al., 1992 ; Allison et al., 2002; Calder et al., 2002, 2007; Pageler et al., 2003; Conty et al., 2007; Hoffman et al., 2007; Carlin et al., 2011 [single cell recordings from macaque]), *the fusiform gyrus* (Wicker et al., 1998; George et al., 2000; Pageler et al., 2003), *the medial prefrontal cortex* (e.g., Calder et al., 2002; Conty et al., 2007), *the orbitofrontal cortex* (see Conty et al., 2007; Senju and Johnson, 2009) and* the amygdala* (Senju and Johnson, 2009)^1^. The definition of ToM is currently under development; while the macro results are not questioned, the micro explanations are still unclear (Schaafsma et al., 2015). For a review of research on the neural mechanisms in face and gaze perception, see Emery (2000), Haxby et al. (2000), Itier and Batty (2009, pp. 849–857), Nummenmaa and Calder (2009), as well as Senju and Johnson (2009).

Could the perceived face and gaze direction be related to fundamental emotional reactions such as fear or fight or, on the positive side, empathy and sympathy? When it comes to the question of how the different neural structures listed above interact during eye contact, Senju and Johnson (2009) explain the eye contact effect through a so called fast-track modulator model (Johnson et al., 2015): according to this model, there is a fast and unconscious subcortical route of face and gaze information (the fast track modulation route) including the superior colliculus, pulvinar, and amygdala (Laeng et al., 2010). Activity in this pathway mediates the affective valence of the stimulus by amygdala-activation and activation of the orbitofrontal cortex. The existence of such a pathway is supported by evidence from research in human newborns (Johnson et al., 2015), as well as from research on patients with cortical blindness (resulting from destruction of the primary visual cortex); despite this loss of primary visual cortex, some patients show activation of the amygdala as a response to facial and bodily expressions of emotion (Burra et al., 2013). In addition to the fast-track-modulation, there is a slow information processing path running through the lateral geniculate nucleus, visual cortex/lateral occipital cortex and inferior ventral temporal cortex, where the gaze direction is decoded by the anterior superior temporal sulcus, and face identity in the fusiform face area. The intentionality of gaze is monitored by the posterior superior temporal sulcus (Nummenmaa and Calder, 2009; Senju and Johnson, 2009). There is also a top-down modulation of the aforementioned anatomical structures from the dorsolateral prefrontal cortex (Senju and Johnson, 2009, p. 130).

Recent research indicates that the amygdala stands central in the analysis of gaze direction (Kawashima et al., 1999; Hoffman et al., 2007; Straube et al., 2010; Boll et al., 2011; Sauer et al., 2014). The function of the amygdala in gaze processing is far more complex than previously suggested: much research has explored which gaze direction leads to the greatest activation of the amygdala. While Kawashima et al. (1999) and George and Conty (2008) found an increased amygdala activation as response to direct gaze, recent studies report an increased amygdala activation to averted vs. direct gaze (Straube et al., 2010) in spite of variation in emotional valence of the faces; moreover, it has been demonstrated that different sub-regions of the amygdala (Boll et al., 2011) are activated differently as response to gaze direction, head orientation, and the valence of facial expression. Sauer et al. (2014) reports that averted vs. direct gaze activates the right dorsal amygdala independent of facial expression and head orientation, while valence effects activated the ventral amygdala, strongly dependent of the direction of head. The left ventral amygdala was strongly activated as response to angry and neutral vs. happy faces when the faces were directed toward the beholder. In the averted head condition, there was an increased activity to happy vs. angry and neutral faces in the left ventral amygdala.

We present three experiments on the effects of face orientation and gaze direction on the judgments of social attributes. As the methods are very similar across experiments, we present them for the sake of simplicity for all experiments together. The same is done for the results for the analysis of association and the inference statistics for all experiments.

## Materials and Methods

In order to test how subjects react to different face and gaze directions, we conducted three experiments in which direction of face and gaze were manipulated: (1) Dutch photographs deriving from the Dutch Radboud Faces Database (RaFD: Langner et al., 2010); (2) Brazilian photographs from the Brazilian FEI face database; and (3) two separate independently repeated experiments using the Holy Face and secular portraits.

A few words about the two different face databases are required. The RaFD^2^ contains colorful pictures of eight emotional expressions: *anger, disgust, fear, happiness, sadness, surprise, contempt* and *neutral*. It consists of “face stimulus sets in which special facial characteristics are systematically varied while other important picture characteristics are kept constant; all frontal images were rated according to the shown facial expression, intensity of expression, clarity of expression, genuineness of expression, attractiveness and valence of expression” (Langner et al., 2010). Hence, this database not only presents neutral expressions, as those selected for in our study, but also a huge spectrum of emotional valence.

The FEI Face Database^3^ has 14 images for each of 200 individuals, a total of 2800 images. These are all in color and taken against a white homogenous background in an upright frontal position with profile rotation of up to about 180° (see Thomaz and Giraldi, 2010, for further information).

In our Experiments 1 and 2, we made a selection of faces with no concern for their emotional ratings (see RaFD database as described by Langner et al., 2010), neither have we transformed the FEI faces in any way (see Thomaz and Giraldi, 2010). In Experiment 1 and 2 the gaze direction in profile direct was manipulated in Photoshop^4^. Frontal indirect/averted faces in Experiment 1 were derived from non-manipulated photos from the RaFD database. Since, in the FEI database, the frontals with averted gaze were not satisfactorily averted for our purpose, they were manipulated in Photoshop.

In the study of the Holy Face, all faces were derived from non-manipulated original paintings. One obvious and essential difference between modern photographs and the painted portraits is that the portraits were painted with intent to present not only a likeness to the physical model, but also to convey a positive response towards the subject of the image. We have chosen to keep the artwork intact because we do not want to interfere with the artist’s design (see Bullot and Reber, 2013a). Each study is evaluated formally by statistical hypothesis testing of our planned contrasts. We explore the structure in the data using a graphical analysis of association. But first we present the material used in the studies.

The advantage of using modern portraits lies in experimental control. In photographs of both frontal and half-profile views, gaze directions can be manipulated. We realized a full 2 × 2-factorial design with two independent factors (face and gaze), resulting in four conditions: frontal view, direct gaze, frontal view, averted gaze, half-profile, direct gaze, half-profile, averted gaze (see Table 1).

**Table 1:** **These sketches illustrate the experimental manipulations in each study (1, 2 and 3)**.

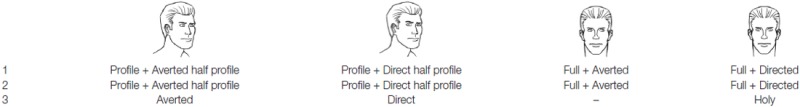

**Figure 1:**
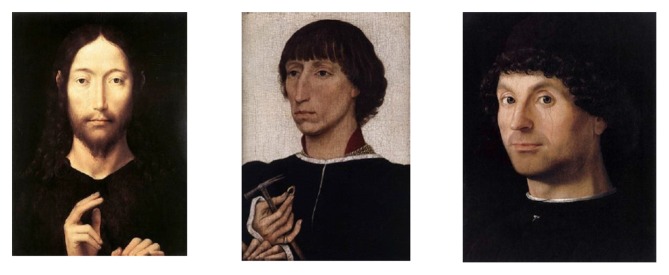
**From left: Holy Face frontal view direct gaze, secular half-profile averted gaze, secular half-profile direct gaze**.

In order to gather information about possible responses to holy vs. profane faces in the 15th century, we decided to examine responses to reproductions of real portraits (see Bullot and Reber, 2013a; Silvia, 2013; but see Graham, 2013; and Bullot and Reber, 2013b; for further discussion). In addition to the first two studies, using material from face databases, a third experiment therefore examined the same responses with reproductions of paintings from the 15th century (Figure 1). Half of the portraits depicted the face of Christ while half of the paintings represented profane faces. As paintings of Christ in frontal view with *averted* gaze were not available to us and we decided to use originals only, the design remained incomplete, and face orientation is confounded with holiness.

**Figure 2:**
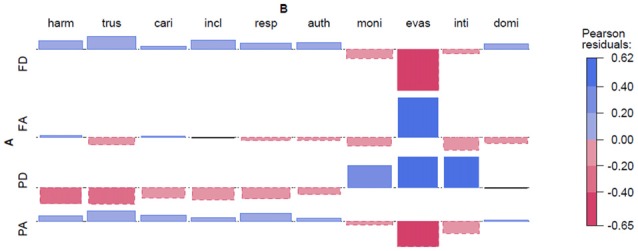
**Dutch Radboud Faces Database (RaFD).** Abbreviations: FD, Frontal Direct; FA, F. Averted; PD, Profile Direct; PA, P. Averted; harm, harmonious; trus, trustworthy; cari, caring; incl, inclusive; resp, respectable; auth, authoritarian; moni, monitoring; evas, evasive; inti, intimidating; domi, dominant. Note: Each Pearson residual is represented by its area; therefore, no *y*-axis is depicted.

Ten adjectives were selected as relevant for inferred personality traits in the collection of Renaissance portraits that is the subject of the investigation on the Holy Face. These adjectives all denote mental traits that are hard to observe directly and objectively. At the same time, these adjectives are common, familiar and often applied as descriptions of the perceived persona that we hypothesize can be inferred from *how* a person looks at an observer. The adjectives can be divided into two different groups. The positive group consists of *“harmonious”, “trustworthy”, “caring, ”inclusive" and “respectable”* and the negative group consists of *“authoritarian”, “monitoring”, “evasive”, “intimidating” and “dominant”*. Some of the adjectives have been used in other related research, see for instance (Todorov et al., 2008b; Figure 2), where the *trustworthiness* dimension is plotted along the *x*-axis and *dominance* along the *y*-axis. All of our adjectives are treated statistically as random factors, i.e., there could be other sets of adjectives that can be used to describe such inferred personality traits. We do not assume that these are the only relevant adjectives for the purposes of our study, but we assert that they are a relevant selection. In an item analysis (see later hypothesis testing) it is possible to estimate whether the items are applied consistently. If the item analysis turns out non-significant, it is indicated that there is a high level of variance, or insecurity, in which adjectives are applied to the experimental material. A significant item analysis confirms that the items are used consistently in line with the results. Four experiments were conducted using ratings on a scale from 0 to 10 on how well an adjective fits an image.

### Methods Specific to Experiments

#### Experiment 1

In Experiment 1 we used photographs from the Dutch RaFD. Fifty-two respondents (36 females and 16 males) volunteered and completed the study. For each participant who completed the study, 20 Norwegian Crowns (about $3) were given to a charity organization for children in need. Each participant received information on the principle of voluntary participation, including the right to withdraw from the study at any time without having to justify their reasons for withdrawal, and all were informed that all data is kept anonymous.

In order to secure the anonymity of older participants who were few in number, we asked participants for age ranges (18–22, 23–27, etc., up to 57 and older). Most participants were 27 years old or younger (*n* = 36), the other participants were evenly distributed across the age ranges older than 27 years. Experiment 1 collected 2080 observations from 52 subjects. Face orientation in Experiment 1 was either frontal or half-profile (45°).

#### Experiment 2

In *Experiment 2* we used a photograph collection of Brazilian faces deriving from the FEI face database. This experiment was essentially identical to Experiment 1, although we used pictures from a different database. Images in Experiment 1 and 2 thus come from two different populations, with slight unplanned differences in face shape and skin tone. Images from the Dutch database are arguably closer to a prototypical Norwegian face. We cannot exclude that this gives rise to familiarity effects. Face orientation in Experiment 2 was either frontal or half-profile, slightly more towards frontal than in Experiment 1.

Forty-nine respondents (39 females and 10 males) completed the questionnaire. Most participants were 27 years old or younger (*n* = 39), the other participants were evenly distributed across the age ranges older than 27 years, similar to Experiment 1. The material was thus similar to study one, except that the models were collected from the Brazilian database. The procedure was identical to Experiment 1. Experiment 2 collected 1960 observations from 49 subjects.

#### Experiments 3 and 4

In *Experiment 3* we used a sample of Renaissance paintings, where the faces represent a profile direct gaze, a profile averted gaze and a holy face with a full face frontal direct gaze (Figure 1). Experiment 4 is an independent replication of Experiment 3. Experiment 3 consists of 1980 observations of how 66 subjects applied the ten adjectives. Experiment 4 consists of 2430 observations of how 81 subjects applied the ten adjectives. The procedure was identical to the first two experiments.

In all experiments the participants gave their active, informed consent by providing their e-mail address, whereupon they received a hyperlink to an online questionnaire hosted by SurveyXact.

### Overview on Statistical Analysis Strategy

#### Analysis of Association Between Image Types and Perceived Personality Traits

Our experiments resulted in measurements for 10 adjectives that denote personality traits. The measurements are divided up by the type of Image that the traits were assigned to by the subjects in the experiments. For the experiments on the photographs from the Brazilian (FEI Database) and Dutch (RaFD) databases, the images were classified into four different categories based on Frontal or Profile view with a Direct or Averted gaze. The categories are abbreviated FA, FD, PA and PD. In the investigations on the Holy Face, there are only three categories: Holy, Direct and Averted (see Table 1 and Figure 1 above). Thus we have many combinations of Image types and Traits to consider, and we will use the mean raw score of assignments in each combination to illustrate how images are associated with traits. One intuitive technique is the Cohen-Friendly Association Plots (Cohen, 1980; Friendly, 1992) as implemented in the program package R. Each plot indicates the deviations from statistical independence of rows and columns in a matrix. Each image category has an indicated line that marks statistical independence, and deviance is marked by boxes that could either be higher (shaded in blue above the line) or lower (shaded in red below the line) than expected from statistical independence. The association graph makes it easy to spot which adjectives are positively or negatively associated with each image type. We decided to use extended association plots with color coded Pearson Residuals (Meyer et al., 2003).

It must be stressed that the association plots are not used as a formal hypothesis test, but rather to illustrate the structure in the data set, and to help us understand the results of the inference statistic. We also want to confirm the quality of the experiment by investigating how the different adjectives contribute to relevant observed differences in the experiments. Additionally, we want to confirm that positive and negative adjectives are assigned differently to the image categories, and thus confirm the validity of the experimental model.

#### Inference Statistics

Assuming that we have found adjectives that correctly associate with positive and negative value assignment, we can make this assignment explicit by multiplying the ratings for the negative adjectives with a constant −1. The assumption is confirmed by analysis of association. If positive and negative adjectives are assigned at random (i.e., unsystematically), we expect the values to sum near zero, i.e., a neutral evaluation on average. However, we can also fail to detect differences between the experimental factors when there is another constant bias (i.e., if all or most images receive a score that deviates from zero by a constant amount, either positively or negatively) resulting in no differences in our experimental conditions (face and gaze direction).

Deviance from any constant assignment can be detected by statistical methods. We have chosen a mixed effects model with random effects for subjects and adjectives. The large number of observations motivates the use of this fairly robust model, as the responses for each adjective are close to normal distribution.

We analyzed all experiments using a mixed effects model implemented in the LmerTest package (see Schaalje et al., 2002; Kuznetsova et al., 2015) in the R statistics software (R Core Team, 2015). The LmerTest implements the Satterthwaite approximation of degrees of freedom, and uses this to evaluate and present the statistical model. This makes it possible and feasible to test a planned model that also includes interaction effects for each experiment, given that there are enough data points to successfully estimate the needed parameters. Previously it was common to perform model comparisons to determine the effect of each experimental factor. The model comparison procedure was awkward and it typically did not provide the degrees of freedom of the final test (see Baayen, 2008, section 7.2). This in turn made it cumbersome to look further into the subject (F1) and item (F2) analyses in random effects models (see Raaijmakers et al., 1999, for a discussion on F1, F2 and alternatives). In this article, the F1 and F2 provide useful insights into the robustness of our findings.

We used random factors for subject and adjectives, thus assuming that there could be other subjects and other adjectives than those we have included. The sufficiency of the chosen adjectives is indicated by how consistently subjects associate the adjectives to the image categories, both within experiments and between repetitions of the experiment. This requires a subject analysis and an item analysis, where we may have a more specific *slope (i.e., rate of change between conditions)* for either subjects or adjectives. Adding specific slopes adds details to the model and reduces the degrees of freedom that are available for random variance. If the model is too complex for the data, we test the model without a specific slope. When a model uses the slope parameter (see formulas below) it indicates that there are not only different starting points (*intercepts*) for each subject and item, but also that the model considers reactions to test images that are specific to the subject or the adjective.

For Experiment 3 and 4 we built models where we explain scores by the type of image (*holy, direct, or averted*) as a fixed factor. The model labeled F1 corresponds to a more detailed *subject analysis*, and the model labeled F2 corresponds to a more detailed *item analysis*. The statistics program uses an estimation procedure that may fail if the model is too complex for the available data. The procedure is therefore to start with the full model, and if the estimation procedure fails then a simpler model will be tried that does not estimate specific random effects called *slopes*, such as type per subject in the formulas below. All models converged.

F1 = score ~ type + (type | subject) + (1 | adjective)

F2 = score ~ type + (1 | subject) + (type | adjective)

For Experiment 1 and 2 we built similar models, but we include gaze and face direction as two fixed factors. We include slopes for all combinations of gaze and face direction for either the subject or the adjective. All these models also converged.

F1 = score ~ direction * gaze + ((direction * gaze) | subject) + (1 | adjective)

F2 = score ~ direction * gaze + (1 | subject) + ((direction * gaze) | adjective)

## Results

### Analysis of Association Between Image Types and Perceived Personality Traits

First, we investigate the structure of the data sets, using extended association plots with color coded Pearson Residuals (Meyer et al., 2003). Each box in the graphs encodes the size of the Pearson Residual for that combination of adjective and type of stimulus. The height of the box indicates deviance of the sum of points, for that combination, from the expected sum if adjectives and types are statistically independent (i.e., the familiar Observed—Expected from a standard chi-square test). The base of each box indicates the square root of the expected sum for that cell. The similar base in all the graphs, thus shows that the expected sums are overall similar, i.e., we have approximately equal evidence from all the adjectives and types of stimuli. The Pearson Residual of a cell is simply the square root of the contribution to significance for that cell in a standard cross tabulation chi-square test. The contributions of the individual Pearson Residuals in the presented graphs are not significant by themselves, which supports that the effects we will see later are not driven entirely by any one adjective.

In the following, the graphs will have four-letter abbreviations for each adjective. The first five adjectives are positive adjectives (*harmonious, trustworthy, caring, inclusive, respectable*). The last five adjectives are negative adjectives (*authoritarian, monitoring, evasive, intimidating, dominant*).

### Experiment 1

Dutch Photographs (see Figure 2). We can see that Front Direct and Profile Direct differ in scores for positive and negative adjectives. Likewise, Profile Direct and Profile Averted are close mirror images of each other. This shows that both face direction and gaze direction plays a role in the assignment of personality traits to the photographs. However, we can also note that the largest effect stems from the adjective *evasive*, which is the adjective that may be the easiest to associate with an averted gaze. It can be noted that there are somewhat larger effects for negative adjectives, although mostly driven by evasive^5^.

### Experiment 2

**Figure 3:**
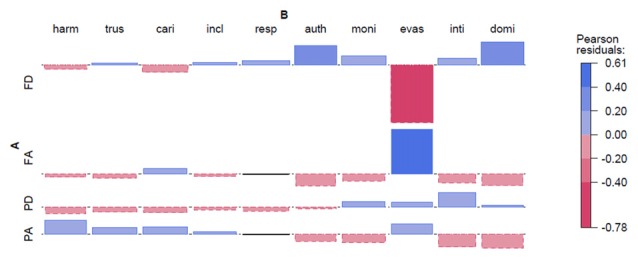
**Brazilian photographs from the FEI face database.** Abbreviations: FD, Frontal Direct; FA, F. Averted; PD, Profile Direct; PA, P. Averted; harm, harmonious; trus, trustworthy; cari, caring; incl, inclusive; resp, respectable; auth, authoritarian; moni, monitoring; evas, evasive; inti, intimidating; domi, dominant. Note: Each Pearson residual is represented by its area; therefore, no *y*-axis is depicted.

Brazilian photographs from the FEI face database (see Figure 3).

The results are very similar to Experiment 1, in that the effect is driven mainly by the adjective *evasive*. There are similarly larger effects for the negative adjectives. We see a tendency for FD/FA and PD/PA to be mirror images of each other, again showing that there is a consistent assignment of personality traits to these photographs as well.

### Experiment 3

**Figure 4:**
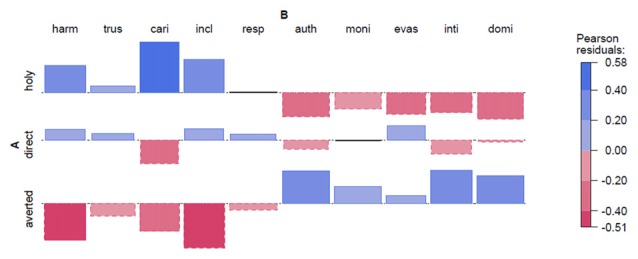
**Holy Faces and secular portraits (1).** Abbreviations: FD, Frontal Direct; FA, F. Averted; PD, Profile Direct; PA, P. Averted; harm, harmonious; trus, trustworthy; cari, caring; incl, inclusive; resp, respectable; auth, authoritarian; moni, monitoring; evas, evasive; inti, intimidating; domi, dominant. Note: Each Pearson residual is represented by its area; therefore, no *y*-axis is depicted.

Holy Faces and secular portraits (1). Figure 4.

In the Experiment on the Holy Face we see a clear tendency for the Holy Face to be associated positively with positive adjectives and a negative association with negative adjectives. The Profile with averted/indirect gaze shows a mirror image of the assignment of traits, compared to the Holy Face. The Profile with direct gaze shows an intermediate profile, with lower effects in general. Importantly, the effects are not driven by any single adjective.

### Experiment 4

**Figure 5:**
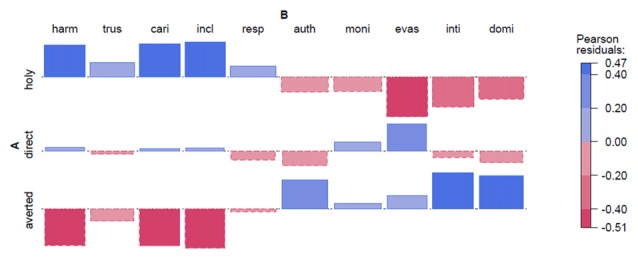
**Holy Faces and secular portraits (2).** Abbreviations: FD, Frontal Direct; FA, F. Averted, PD; Profile Direct, PA, P. Averted; harm, harmonious; trus, trustworthy; cari, caring; incl, inclusive; resp, respectable; auth, authoritarian; moni, monitoring; evas, evasive; inti, intimidating; domi, dominant. Note: Each Pearson residual is represented by its area; therefore, no *y*-axis is depicted.

Holy Faces and secular portraits (2) (see Figure 5).

This Experiment replicates all the findings in Experiment 3 for associations between adjectives and personality traits in the collection of portraits. This importantly shows that the results are robust and likely to replicate well.

### Analysis of Association Between Image and Personality Traits

Experiment 1 and 2 using photographs, show that subjects are indeed very consistent in how they assign personality traits to images. However, it seems that differences between the experimental conditions mainly stem from the application of the adjective *evasive*, which is also the adjective that may more easily be associated with the lack of direct eye contact through plain observation.

Experiment 3 and 4 show something that is a little more interesting. Again the subjects are very consistent in how they assign the adjectives, but this time there is a noticeable difference between the experimental conditions when it comes to assigning positive or negative traits to the portraits. This effect is not driven by any particular adjective, but applies across all adjectives.

One main difference between photographs and the portraits is that the portraits are constructed by an artist, with the intention to present a persona. Reflecting on the differences between the photographs and the portraits it should be noted that they differ in many dimensions. The portraits include mainly men at the peak of their careers, whereas the photographs are generally of younger individuals, and include both men and women, as well as ethnic differences noticeable from for example skin tone and face shape.

There is obviously a need to follow up these differences in controlled experiments, rather than speculating *post hoc* on how other factors contribute to our perception and assignment of personality traits. It is clear that our subjects are very consistent in their judgments, but it is also clear that there are other factors to consider, such as age and social class of the person depicted in the pictures.

We are now ready to move on to hypothesis testing using inference statistics. We have shown that assignment of personality traits to images are consistent and related to our experimental conditions, but are these findings significant, or can they be explained by the variance in our samples?

### Inferential Statistics

We start with the analysis of the photographs for Brazilian and Dutch faces. The regression model shows the intercept, and the effects of profile, indirect gaze and the interaction effect of both profile and indirect gaze. The intercept is positive for both experiments, but this is not significant when tested. The main effect of face direction is negative (Brazilian: −0.15, Dutch: −0.74) for profile, which is significant for the Dutch set only (F1: **p < 0.002, F2: *p < 0.03). The main effect of gaze direction is negative for indirect gaze (Brazilian: −0.05, Dutch −0.35), which is significant for the Brazilian and Dutch subject analysis (*p < 0.05 and **p < 0.01, respectively), but not in the item analyses. The interaction effect shows a more positive effect for profile with an indirect gaze for both Brazilian and Dutch sets (accounts for 0.36 and 1.05 points respectively), which is significant for subject analysis only (*p < 0.02 and ***p < 0.001, respectively). The failures to reach significance for the item analyses show that there is considerable variance for how the adjectives affect ratings for the photographs, and this was previously (see Figures 2, 3) identified as an effect driven by a few adjectives, *evasive* in particular.

**Figure 6:**
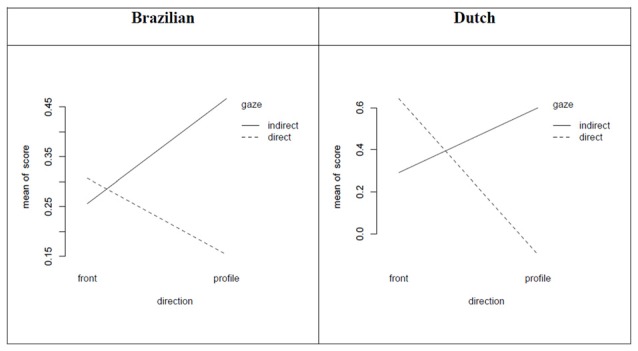
**Interaction between face and gaze direction for the Brazilian and Dutch faces (Experiment 1 and 2)**.

The regression formulas for the Brazilian (a) and Dutch (b) study are presented below. For estimates of the values for each combination, see the interaction graph (Figure 6).

(a) points = 0.31 −0.15(if *profile*) −0.05(if *indirect gaze*) + 0.36(if *profile* and *indirect gaze*)
(b) points = 0.64 −0.74(if *profile*) −0.35(if *indirect gaze*) + 1.05(if *profile* and *indirect gaze*)

Figure 6 shows the net result of all combinations of face and gaze direction. Experiment 1 and 2 look similar. In both datasets, the profile that looks directly at the viewer has the lowest score. In both datasets the scores are slightly biased towards positive scores. Frontal direct gaze has a more positive score, which is more noticeable in the experiment on Dutch faces. Both experiments show interaction between face and gaze direction, but there is considerable insecurity in the assignment of scores, and with the exception of Dutch face direction, the item analysis is not significant. This indicates that although subjects assign positive and negative adjectives consistently there is little agreement for when a specific adjective is used, and some adjectives such as “evasive” contributes more to the effects.

### The Studies on the Holy Face (Experiments 3 and 4)

The following table gives the results of the statistical analysis of Experiment 3 and 4, relating to painted portraits. Holy1 and Holy2 are independent replications of the same experiment. As can be seen from Table 3, and the point calculations below, there are significant differences in the application of positive and negative values between the three variants of portraits. The portraits with a direct gaze are neutral (at a non-significant intercept of −0.22 and +0.007, respectively). The Holy Face is more positive (0.45 (i.e., +0.67 −0.22), and 0.44 (i.e., +0.37 +0.07) points respectively). A face with an averted gaze is more negative (−0.88 (i.e., −0.66 −0.22), and −0.77 (i.e., −0.78 +0.007) respectively). The expected distance between the Holy Face and the averted profile is about 1.2 points (1.33 and 1.15, respectively). The ANOVA model tests that the effects for type of portrait are persistent across both subject and item analyses. Significance tests reveal 2–3 stars for all relevant comparisons. Holy2 almost perfectly replicates Holy1. This means that the observed differences in adjective assignment are very likely to be true differences. The *Direct* images are equal to the intercept, as neither *Holy* nor *Averted* is added in the formula.

The regression formulas for the Holy1 (a) and the Holy2 (b) study are presented below.

(a) points = −0.22 +0.67(if *Holy*) −0.66(if *Averted*)
(b) points = 0.007 +0.37(if *Holy*) −0.78(if *Averted*)

**Figure 7:**
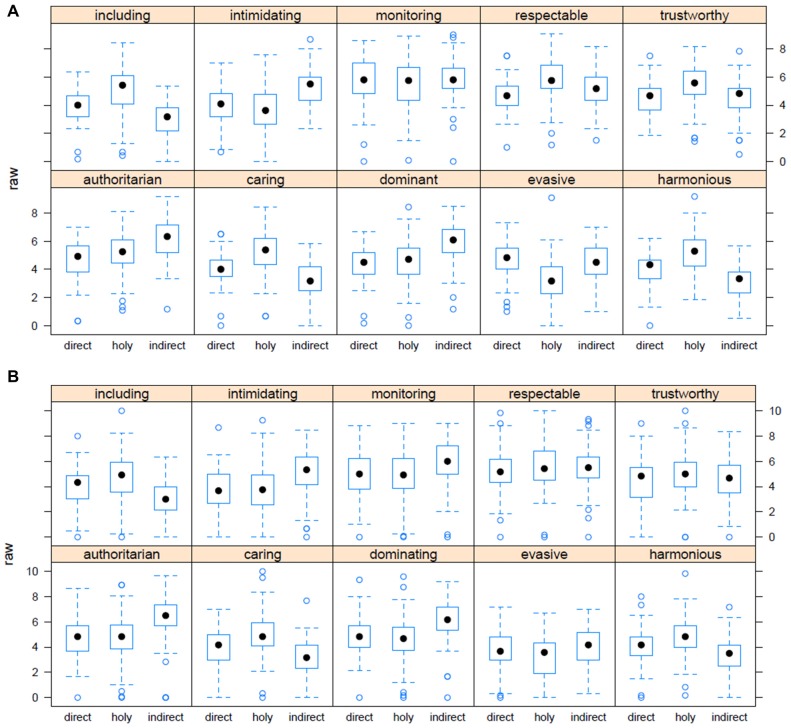
**(A)** How well the adjectives fit with the type of picture. Experiment 3. **(B)** How well the adjectives fit with the type of picture. Experiment 4.

Looking more closely at the relationship between adjectives and type of portrait indicates that Experiment 3 and Experiment 4 are indeed good replications of the same experiment. Figures 7A,B show this for the range of the original data for all adjectives in both experiments. From the analysis of association, we confirmed that the effects are not driven by individual test items, and this shows up here in highly significant item analyses for both experiments.

## General Discussion

The basic motivation of this study was to examine the potential reasons for why Medieval and Renaissance painters depicted Christ with a face shown from a frontal view and looking directly at their viewers but showed portraits of profane sitters from the half-profile. The results with *modern pictures* of faces showed that people gave frontal faces with direct gaze high scores on attributes deemed relevant to divinity (Dutch faces more so than the Brazilian ones), but profile views with averted/indirect gaze were also more positive, as far as the modern faces are being concerned (see Table 2). The findings were even more pronounced for historical portraits of Christ, where the Analysis of Association shows a consistent overrepresentation of positive adjectives for the Holy Faces.

**Table 2:** **Results of statistical analysis of experiment 1 and 2**.

	Brazilian		Dutch	
	Regression model		Regression model	
	Obs.: 1960 subj.: 49 adj.: 10		Obs: 2080 subj.: 52 adj.: 10	
Intercept	0.31		0.64	
Direction: profile	−0.15		−0.74 *t*_(60.6)_ = −6.8 *p* < 0.001***	
Gaze: indirect	−0.05		−0.35 *t*_(87.8)_ = −3.5 *p* < 0.001***	
profile × indirect	0.36 *t*_(53.8)_ = 2.6 p < 0.05*		1.05 *t*_(56.5)_ = 6.8 *p* < 0.001***	

	**F1**	**F2**	**F1**	**F2**

Direction	*F*_(1,60.2)_ = 0.19 n.s.	*F*_(1,10.2)_ = 0.1 n.s.	*F*_(1,255.2)_ = 9.8 p < 0.002**	*F*_(1,12.7)_ = 7 p < 0.03*
Gaze	*F*_(1,57.1)_ = 4.0 p < 0.05*	*F*_(1,9.0)_ = 0.4 n.s.	*F*_(1,1743.3)_ = 6.7 p < 0.01**	*F*_(1,10.4)_ = 3.3 *p* < 0.1
Direction × gaze	*F*_(1,53.8)_ = 6.8 p < 0.02*	*F*_(1,9.1)_ = 1.1 n.s.	*F*_(1,56.5)_ = 47 p < 0.001***	*F*_(1,9)_ = 4.5 *p* < 0.1

*Significance above estimates if the coefficient is different from 0 and t(df) comes from F1. *p*-value (type III, with Satterthwaite approximation for degrees of freedom)*.

The Holy Faces and the Dutch modern frontal faces gazing at the viewer were regarded to be more caring, trustworthy, harmonic, inclusive and respectable. Experiment 1 (Dutch) however, showed similar effects for profiles with averted/indirect gaze (see Figure 2). In Experiment 2 (Brazilian) the most positive combinations were profiles with averted gaze (see Figures 3, 6), and in both Experiment 1 and 2 profiles with direct gaze got a lower score (see Figure 6). Hence, the profiles of modern faces gazing at us were scored more negatively, in contrast to the results on the artworks, where direct gaze was scored as more positive than averted, for the half-profiles. The “eye contact effect” (Senju and Johnson, 2009), in the photographs and in the artworks, is discussed later. Although the effects are more uncertain for Experiment 2 (Brazilian), our findings raise the question whether the frontal Holy Face is more than a convention, that is, whether there are deeper biological or evolutionary reasons for such a convention.

Despite the support of our hypothesis that Medieval and Renaissance artists tried to convey saintly attributes by depicting faces from the frontal view and with direct gaze, our study points to the limits of experimental art history. First, it is impossible in principle to know whether modern viewers provide the same scores as a medieval audience would have done. While the neuroscientific theories of art (e.g., Ramachandran and Hirstein, 1999; Zeki, 1999) claim that the brain has been the same the last millennia and judgments are therefore comparable, proponents of a historical theory of art appreciation emphasize the cultural and experiential differences between medieval and modern people, which renders comparison difficult (see Bullot and Reber, 2013a). The more closely the scientific hypothesis is related to the biological perceptual system, the more we can generalize results across time. For example, we might assume that the biological basis of color vision or perception of movement did not change across time. However, most hypotheses, and presumably the most interesting ones, must take art-historical knowledge (see Bullot and Reber, 2013a) into account, rendering such generalization more difficult.

Second, both modern faces and historical materials have their advantages and disadvantages. As there are face databases with portraits from different angles, and as gaze direction is relatively easy to change by technical means, experimental control of the materials is possible. Moreover, with such materials, we can remove much of the influence of cultural knowledge on the judgments. This strength turns into a weakness, however, if cultural knowledge moderates the effects of facial features. For example, knowing how Christ is depicted might provide different or—as in our study—stronger interpretations of facial orientation and gaze direction. Modern materials may be inadequate substitutes for the historical paintings. However, the latter are problematic, too. As there are few depictions of the Holy Face from half-profile and almost no depictions of profane frontal faces, experimental manipulation of materials is difficult, especially as the manipulation of face orientation would demand building a 3D model from one image in profile. Some of the effects observed in the last study could stem from material effects; for example, that profane people are perceived to be more dominant and intimidating may derive from the perceived age of the depicted person, an assumption that awaits further research.

Moreover, we must take into consideration that there are some physiognomic traits that are perceived to be more trustworthy or dominant (Oosterhof and Todorov, 2008; Todorov, 2008; Todorov et al., 2008a,b; Geniole et al., 2014) and intelligent (Kleisner et al., 2014) than others (for a recent review and discussion of this field of research: Todorov et al., 2015). In our study, we had no possibility to control for such parameters. Neither did we manipulate the Renaissance portraits into neutral expressions; hence, where the photos from the databases were displaying the same neutral mouths and eyes, the art portraits vary to a certain degree from neutral expressions.

We consider evaluating the materials on a perceived gender scale. As has been demonstrated by Todorov et al. (2015, p. 522) and Figure 2, perceptions of dominance are strongly related to masculine appearance. The Holy Face is sometimes feminine in its beauty. This is particularly interesting when it comes to the judgment of aggression, which strongly correlates with the facial width-to-height ratio in male faces; increasing the ratio leads to stronger judgments of aggression (Geniole et al., 2014).

When it comes to the question of symmetry vs. non-symmetry in faces, only the Holy Faces are strictly symmetrical. It has been demonstrated (Ewing et al., 2010) that frontal faces with direct gaze are rated to be more attractive than those with averted gaze. Turning the face upside down did not, however, confirm the results, which indicate that the symmetry of faces does not explain the full effect. On the other hand, the so called Thatcher Illusion demonstrates that perception of facial features is generally suppressed if a face is inverted (Thompson, 1980).

Problematic for the interpretation of our results that a representation of Christ as God, for bio/evolutional reasons, *had to be* represented symmetric is the fact that the Byzantine Christ the Pantocrator (All Ruler), in many cases, is not symmetric, combining profile and full-face, particularly conspicuous in the Deesis mosaic (*ca.* 1300) on the Gallery of Hagia Sofia, Istanbul^6^. Nevertheless, this “manipulation” with the physiognomy is so subtle that one may have to scrutinize the face to find out how asymmetric it really is. On the other hand, the Byzantine so called Mandylion (Nicolitti, 2014 inter al.), said to be an imprint of Christ’s face, as in the veil of Veronica, is as precisely symmetric as the Holy Faces of Western Renaissance. Hence, in all these cases, Christ is represented full-face, in the Western Renaissance with a direct gaze, in the Mandylion with a direct or weakly averted gaze, in the Byzantine Pantocrator more or less with a direct gaze, but also sometimes averted (a famous example of averted gaze being the Pantocrator in the cupola of the monastic church of Dafni, Greece (early 12th century).

Experimental art history, like experimental archeology, can only test the plausibility of assumptions. Even if we could assume that judgments from modern respondents correspond to those by historical respondents, in that the attributes of modern materials map the attributes of historical materials and that historical materials yield reliable and valid responses, we still cannot prove that painters indeed intended to achieve the effects that were obtained with modern viewers. In general, experiments to test hypotheses about the intended or real effects of historical art on the audience of that time are of some merit and might provide us with insights about the plausibility of art-historical assumptions. However, the interpretation of such studies always has to take change in cultural knowledge of the audience into account.

In this study, we have seen that frontal faces with direct gaze (Experiment 1, 3 and 4) were more highly associated with positive adjectives. The exceptions from Experiment 2, and the increased insecurity in that experiment, might be explained by the fact that the photographs included more factors such as gender and ethnicity that was not an issue in the painted portraits, as argued earlier in the results section on the Analysis of Association. Does this also help to associate positive values to the Holy Face in a Western context? This raises the question about the depiction of Christ as a white Western man. It looks like the explanation for our readiness to apply positive values to the Holy Face might be a combination of many factors. Familiarity may be such a factor as familiarity will signify a more typical face, and it is found to be rated as more trustworthy than an extremely beautiful face (Sofer et al., 2015).

Face and gaze direction might reinforce the general impression. There could be other factors that influence our perception of beauty that work in the same direction, such as symmetry and possibly androgyny. That means that several factors may conspire together towards a positive perception of the image. In order to explore these factors in more detail and how they influence the cognitive processing of faces, neuroimaging studies may be applied that address the differential processing of face and gaze direction on a neuronal level. This would also open the possibility for exploring the underlying mechanism from a more implicit perspective, i.e., without directing the subject’s attention onto possible attributes of the perceived face. This would correspond to a more natural and ecologically more valid observation situation.

The Holy Faces were perceived as particularly *caring, inclusive* and *harmonious*, but also *trustworthy* and *respectable*. This strong bias towards the positive adjectives will partly seem to find its explanation in cultural factors: that the face of Christ will be associated with such positive adjectives. Still, there is another feature with the Holy Faces that may override the cultural one: many of the Holy Faces appear feminine when the beard is occluded, a feature that has to be followed up with new studies.

In our two studies on the Holy Face, the secular portraits with direct gaze were neutral (see Table 3) and those portraits with averted gaze were negative. This result may be explained through a combination of the eye contact effect (see Senju and Johnson, 2009), and the effect of the general impression of the faces: if the general impression of the face is that it is authoritarian, monitoring and intimidating, this will bias the perception of direct gaze in the negative direction. By contrast, if the face gives a positive impression, this will also strongly interact with the eye contact effect resulting in a bias toward the positive adjectives. The portraits that we use in our survey show different valences in face expression. Most of the faces with direct gaze are close to neutral, but there are also some that are scary and some with inclusive features.

**Table 3:** **Results of statistical analysis of Experiment 3 and 4**.

	Holy1		Holy2	
	Regression model		Regression model	
	Obs.: 1980 subj.: 66 adj: 10		Obs: 2430 subj.: 81 adj.: 10	
Intercept	−0.22 n.s.		0.007 n.s.	
Holy	0.67 *t*_(65)_ = 6.2 p < 0.001***		0.37 *t*_(81.2)_ = 3.0 p < 0.01**	
Averted	−0.66 *t*_(65)_ = −8.3 p < 0.001***		−0.78 *t*_(115.6)_ = −10.3 p < 0.001***	

	Difference from intercept is indicated.			

	**F1**	**F2**	**F1**	**F2**

Type	*F*_(2,65)_ = 75.7 *p* < 0.001***	*F*_(2,9)_ = 25.8 *p* < 0.001***	*F*_(2,121.8)_ = 68.8 *p* < 0.001***	*F*_(2,9.7)_ = 21.7 *p* < 0.001***

*Significance above estimates if the coefficient is different from 0 and t(df) comes from F1. *p*-value (type III, with Satterthwaite approximation for degrees of freedom)*.

To sum up, our study suggests that experimental art history can provide evidence for the plausibility of psychological assumptions that follow universal evolutionary or biological laws. However, cultural and historical factors prevent experimental art history from providing more direct evidence on the processes that guided the generation of art in the remote past.

## Ethics Statement

All research reported in this manuscript was in accordance with the ethical guidelines of the American Psychological Association and the ethical guidelines for the social sciences, law, and the humanities by the Norwegian National Research Ethics Committees.The study was exempt from the requirement to submit it to one of the Norwegian bodies for ethical approval because it was conducted with healthy adults who could provide informed consent for themselves, all data were collected anonymously, and thestudy did not involve invasive methods or other potential harm, deception, potential concerns about privacy, etc.

## Author Contributions

POF: outlining theory, designing experiments, conducting study, writing manuscript. CJ: outlining theory, data analysis, data visualization, writing manuscript. LH and RR: outlining theory, designing experiments, writing manuscript. ØOS: data analysis, writing manuscript. AEA and LCS: preparing materials. KS: writing manuscript.

## Funding

The Meltzer Foundation, LLE at the University of Bergen and NTNU supported the project “Symmetry in Art and Science An interdisciplinary research project”.

## Conflict of Interest Statement

The authors declare that the research was conducted in the absence of any commercial or financial relationships that could be construed as a potential conflict of interest.

## Footnotes

^1^The regions here listed serve different functions: the superior temporal sulcus is considered to be a hub region in social perception and cognition, including language, head and gaze stimuli. It consists of a number of sub regions responding selectively to particular stimuli, organized along a posterior-to-anterior axis (see particularly Deen et al., 2015, for new evidences). The fusiform gyrus responds to face and body stimuli; it has recently been suggested that face and body patterns each add unique information to the response patterns to whole persons (see Kaiser et al., 2014). The medial prefrontal cortex is strongly involved in decision making and in memory processes (Euston et al., 2012). It is also involved in judgments of self and others (Denny et al., 2012), moral judgments, and even aesthetic judgment tasks (Jacobsen et al., 2006; Forbes and Grafman, 2010; Zaidel and Nadal, 2011; Avram et al., 2013). The orbitofrontal cortex receives exteroceptive and interoceptive stimuli; it is a significant part of our reward network, is strongly connected to the emotional network, i.e., the limbic system, and can be considered as a higher order cortex for smell and gustation. It is activated as a response to what a subject finds beautiful either in art or in music (Kawabata and Zeki, 2004; Ishizu and Zeki, 2011). All these areas are involved in many tasks, and may perform sub-functions in an intricate network.

^2^http://preview.tinyurl.com/gp5bpbo

^3^http://preview.tinyurl.com/h6nbsma

^4^The eyes in the databases were not corresponding to the even more averted eyes in the historical material.

^5^It is therefore strange that profile direct (PD) was more evasive than PA. This may be explained by a more general uncertainty for how subjects apply adjectives in Experiment 1 and 2, which is exemplified by the fact that typically the item analyses (see Table 2) do not reach significance.

^6^The asymmetry of Christ’s physiognomic traits in the mosaic in Hagia Sophia corresponds to the prescriptions in the *Greek Painters Manual*, the so called *Long Paragraph* (LP), a canon presented under the name of the Macedonian painter Manuel Panselinos (Torp, 1984, p. 39–40).
